# The Death of Dr. C. R. Coffin

**Published:** 1891-04

**Authors:** 


### Obituary.

### The Death of Dr. C. R. Coffin.

In the death of Dr. C. R. Coffin , of South Kensington, on the 29th of December last, at the age of sixty-five, the profession has lost a valuable worker. Although Dr. Coffin has for several years taken no active part in professional affairs his work and its results still linger in the thoughts of many. A quiet, unobtrusive, but keenly observant and appreciative mind is an adornment, but is not too common. Dr. Coffin will be remembered alike for the purely technical work with which his name is associated, as well as for the earnestness with which he took up and worked in the interest of the British Dental Association. Dr. Coffin was born and educated in the United States, studying his profession at Boston, and obtaining his qualification at Baltimore College in 1853. In 1855 he came to England, at first practicing for a few years in Manchester, and subsequently in London. As is well-known Dr. Coffin made some interesting experiments on the properties of gold foil in removing the excess of mercury from amalgam fillings.—*Dental Record.*

